# A systematic review of the relationships between nurse leaders' leadership styles and nurses' work‐related well‐being

**DOI:** 10.1111/ijn.13040

**Published:** 2022-01-31

**Authors:** Milja Niinihuhta, Arja Häggman‐Laitila

**Affiliations:** ^1^ University of Eastern Finland Finland; ^2^ Social and Health Care, City of Helsinki Helsinki Finland

**Keywords:** leadership style, nurse leader, nurses, systematic review, work‐related well‐being

## Abstract

**Aim:**

This systematic review aimed to summarize current research knowledge about the relationships between nurse leaders' leadership styles and nurses' work‐related well‐being.

**Background:**

Due to the global shortage of nurses, it is essential for nurse leaders to maximize staff retention and work‐related well‐being.

**Methods:**

Following Cochrane Collaboration procedures, the PRISMA statement and PRISMA checklist, relevant quantitative studies published between 1 January 2012 and 31 December 2020 were retrieved from the CINAHL, Scopus, PubMed and Medic databases and then systematically reviewed. Seventeen cross‐sectional and follow‐up studies with surveys were retained for inclusion and evaluated with the Critical Appraisal of a Survey instrument. The data were summarized narratively.

**Results:**

Three core themes of leadership styles: destructive, supportive and relationally focused, were identified, with statistically significant direct and indirect connections between nurses' work‐related well‐being. Well‐being was mainly assessed in terms of burnout. Effects of leadership styles on work‐related well‐being were reportedly mediated by trust in leader, trust in organization, empowerment, work‐life conflict, relational social capital, emotional exhaustion, affectivity, job satisfaction and motivation.

**Conclusion:**

Nurse leaders' leadership styles affect nurses' work‐related well‐being. In developing intervention studies and providing training on work‐related well‐being, the impact of the indirect effects and the mediating factors of the leadership styles should be acknowledged.

## Summary statement

What is already known about this topic?

- Nurse leaders play a key role in maximizing staff retention and influencing healthcare organizations' productivity and effectiveness.
- Nurse leaders' leadership styles strongly affect the nursing staff, their working environment and work‐related well‐being.

What this paper adds?

- This systematic review shows that nurse leaders' destructive, supportive and relationally focused leadership styles significantly affect nurses' work‐related well‐being.
- It provides detailed information regarding direct and indirect associations between leadership styles and work‐related well‐being and the mediating variables for indirect associations.
- The data suggest that nurse leaders should be capable of using supportive and relationally focused leadership styles.

The implications of this paper

- Systematic evaluation of nurse leaders' leadership styles in organizations is important because they significantly affect nurses' work‐related well‐being.
- Results of this review could be used when developing nurse leaders' leadership styles and work environments and planning and implementing leadership training.
- In the developing of intervention studies on work‐related well‐being, the results of the indirect effects and the mediating factors of the leadership styles should be acknowledged.

## 1 INTRODUCTION

The worldwide shortage of nurses (Chan et al., 2013; Heinen et al., 2013; WHO, 2016) poses a major challenge for leadership in nursing. Nurses who stay in their profession, work efficiently and produce good patient outcomes, have also generally high work‐related well‐being (Long, 2020; Nantsupawat et al., 2016; Van Bogaert et al., 2014). Several reviews (Cummings et al., 2018; Skakon et al., 2010; Weberg, 2010) rooted in different disciplines have found that managers' use of certain leadership styles can enhance relationships with employees, performance, productivity, the working environment and work‐related well‐being. Conversely, inappropriate leadership increases costs, employee turnover and absenteeism while reducing performance.

Leaders' well‐being affects that of their subordinates (Skakon et al., 2010). However, previous reviews (Awa et al., 2010; Häggman‐Laitila & Romppanen, 2017; Romppanen & Häggman‐Laitila, 2017; Van Wyk & Pillay‐Van Wyk, 2010; Westermann et al., 2014) indicate that this relationship is poorly studied. Most interventions targeting nurses' and nurse leaders' work‐related well‐being are focused on individuals' cognitive and behavioural skills. The most intensively studied aspects are burnout and stress management, although well‐being is widely understood in broader terms. According to a review by Buffer et al. (2013), work‐related well‐being is a comprehensive concept, which includes (besides occupational health and health behaviour), social and economic well‐being and well‐being connected to professional development, as well as both psychological and physical health. Moreover, each of these aspects are multidimensional. For example, psychological well‐being encompasses self‐esteem, autonomy, personal growth, sense of purpose in life, social support and mastery of environment (Jacobs et al., 2013).

As nurse leaders play key roles in staff retention and both the productivity and effectiveness of healthcare organizations, their performance should clearly be developed using evidence‐based knowledge. Four previous reviews (Adams et al., 2019; Cummings et al., 2018; Long, 2020; Weberg, 2010) have examined effects of nurse leaders' different leadership styles on the nursing staff, their working environment and work‐related well‐being. Long's (2020) review of authentic leadership style showed that authentic leaders can promote newly qualified nurses' work‐related well‐being and retention. Adams et al. (2019) focused on the relationship between nurse managers' role and the well‐being of ICU nurses and found that nurse managers' behaviours affected the well‐being of their subordinates, through for example supportive behaviour, trust and inclusion in decision‐making.

Although these reviews provided valuable insights, systematic reviews are needed to identify leadership styles that maximize nurses' work‐related well‐being and to provide recommendations based on the latest and best research evidence. Such recommendations are needed to support the development of organizational practices and effective healthcare environments and policy. Thus, this article presents a systematic review concentrating exclusively on nurse leaders' leadership styles and nurses' work‐related well‐being. The aim was to summarize empirical research on the relationships between them by addressing three research questions: ‘Which leadership styles adopted by nurse leaders have been studied in connection with nurses' work‐related well‐being?’, ‘How was work‐related well‐being measured in those studies?’ and ‘How did the studied leadership styles reportedly affect nurses' work‐related well‐being?’

## 2 METHODS

### 2.1 Design

This systematic review of quantitative studies was designed and implemented in accordance with Cochrane Collaboration protocols (Higgins & Green, 2011) and the PRISMA statement (Moher et al., 2009, Figure 1).

**FIGURE 1:**
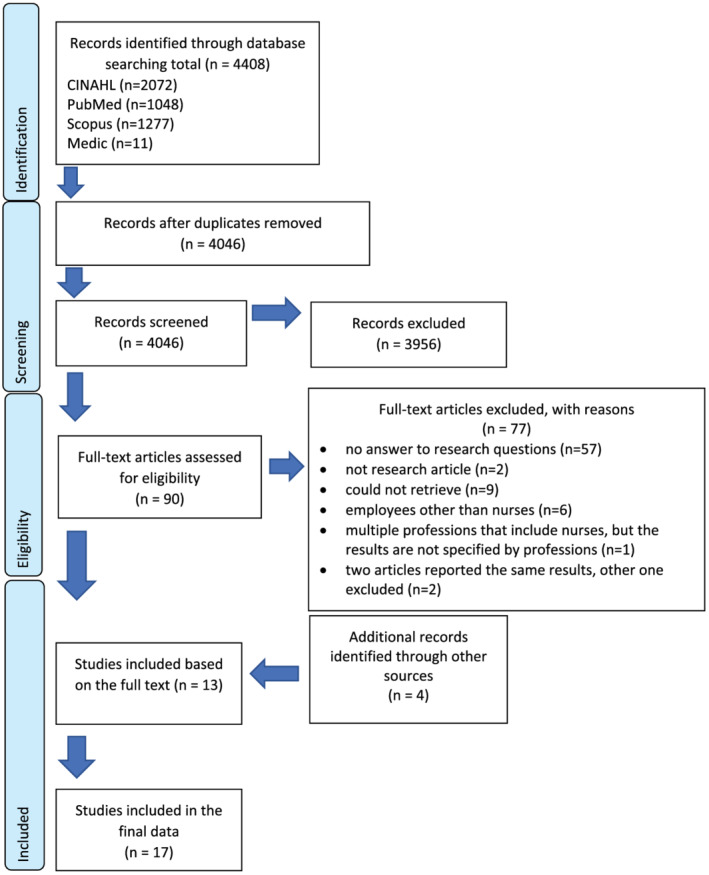
PRISMA flowchart

### 2.2 Search strategy and inclusion criteria

The CINAHL, Medic, SCOPUS and PubMed databases were systematically searched for relevant studies. The search strategies and terms were tailored to each database individually with guidance from a library‐based information specialist. Searches were performed by combining search terms using the Boolean operators AND, OR and NOT. Searches were limited to peer‐reviewed articles in English or Finnish languages published between 1.1.2012 and 31.12.2020 to obtain an updated view to the research topic and to avoid overlapping results with the previous review (Cummings et al., 2018) (Table 1). The inclusion criteria were quantitative studies with experimental, quasi‐experimental or descriptive designs on the relationships between nurses' work‐related well‐being and the support, leadership skills, or leadership style of nurse leaders.

**TABLE 1:** Search terms

Search terms			Limiters
Leader*	Well‐being	Competenc*	2012–2020
Personnel manage*	Wellbeing	Skill*	English
Human resource*	Job satisfaction	Style*	Finnish
Manage*			Peer‐reviewed articles

### 2.3 Search outcome and exclusion criteria

The search yielded 4408 hits: 1048, 2072, 1277 and 11 from the PubMed, CINAHL, SCOPUS and Medic databases, respectively. After removing duplicates (362), 4046 articles remained for title and abstract review, which was performed by two researchers (Figure 1).

Articles retrieved based on abstracts were excluded from further analysis, if they were not written in English or Finnish and/or irrelevant to the research questions. Reviews, editorials and discussion papers were also excluded. After this exclusion process, 90 articles remained for consideration. Two researchers read the full texts of these articles to evaluate their eligibility for the review, resulting in the exclusion of a further 77 articles. Articles were also excluded after examination of the full texts if they did not address the research questions, were not research articles, could not be retrieved, addressed employees other than nurses or multiple professions, but the findings were not separated by professions, or previously retrieved articles reported the same results. Researchers screened every article independently. The disagreements between the researchers were solved through consensus discussions. The third opinion was not needed. The 13 articles remaining after this process were selected for inclusion, and their references were manually screened by two researchers to identify additional relevant articles. As a result, four further articles were included in the review (Figure 1).

### 2.4 Quality assessment

The final data consisted of 13 cross‐sectional and 4 follow‐up studies with surveys. They were assessed by two researchers with the checklist, Critical Appraisal of a Survey, developed for the survey studies (Center for Evidence‐based Management, 2014). It included 12 criteria (Table 2). The checklist did not include any guidance regarding rating the quality of the studies; therefore, the authors decided together the scale of ranking (Baatiema et al., 2017; Bahadori et al., 2020; Protogerou & Hagger, 2019). The researchers decided that if the study met over half of the criteria (7/12), it was accepted for the review. The quality was appraised satisfactory if the study met 7 or 8 of 12 criteria, and good if it met over 75% of the criteria (9 or 10/12). The quality of three studies was deemed satisfactory and others (*n* = 14) had good quality. All the studies that were included in the review addressed clearly focused questions using appropriate research methods, and the method used to choose subjects was described clearly. All the studies used representative samples and trustworthy measurement instruments. Satisfactory response rates were obtained in 14 of the studies, and the statistical significance of the results was assessed in all of them. However, confidence intervals were not reported in 11 of the articles, the minimum required sample size was only determined by preliminary statistical power analysis in six of the studies, and 16 of the articles did not address the possibility of confounding factors. All studies yielded results that were applicable to the authors' organization (Table 2).

**TABLE 2:** Critical appraisal questions for the survey

Appraisal questions	Article																
	Bobbio et al. (2012), Italy	Bobbio and Manganelli (2015), Italy	Ebrahimzade et al. (2015), Iran	Kaffashpoor and Sadeghian (2020), Iran	Laschinger et al. (2013), Canada	Laschinger et al. (2014), Canada	Laschinger and Fida (2014), Canada	Laschinger et al. (2015), Canada	Majeed and Fatima (2020), Pakistan	McKenna and Jeske (2021), Ireland	Munir et al. (2012), Denmark	Nelson et al. (2014), Canada	Pishgooie et al. (2018), Iran	Read and Laschinger (2015), Canada	Rodwell and Munro (2013), Australia	Sabbah et al. (2020), Lebanon	Trepanier et al. (2019), Canada
1. Did the study address a clearly focused question/issue?	Yes	Yes	Yes	Yes	Yes	Yes	Yes	Yes	Yes	Yes	Yes	Yes	Yes	Yes	Yes	Yes	Yes
2. Is the research method (study design) appropriate for answering the research question?	Yes	Yes	Yes	Yes	Yes	Yes	Yes	Yes	Yes	Yes	Yes	Yes	Yes	Yes	Yes	Yes	Yes
3. Is the method of selection of the subjects (employees, teams, divisions, organizations) clearly described?	Yes	Yes	Yes	Yes	Yes	Yes	Yes	Yes	Yes	Yes	Yes	Yes	Yes	Yes	Yes	Yes	Yes
4. Could the way the sample was obtained introduce (selection) bias?	No	No	No	No	No	No	No	No	Yes	Yes	No	No	No	Yes	No	No	No
5. Was the sample of subjects representative with regard to the population to which the findings will be referred?	Yes	Yes	Yes	No	Yes	Yes	Yes	Yes	Yes	Cannot tell	Yes	Yes	Yes	Yes	Yes	Yes	No
6. Was the sample size based on pre‐study considerations of statistical power?	No	No	Yes	Yes	No	Yes	No	No	Yes	No	No	No	Yes	No	No	Yes	No
7. Was a satisfactory response rate achieved?	Yes	Yes	Yes	Yes	Yes	Yes	Yes	No	Yes	Yes	Yes	No	Yes	Yes	Yes	Yes	No
8. Are the measurements (questionnaire) likely to be valid and reliable?	Yes	Yes	Yes	Yes	Yes	Yes	Yes	Yes	Yes	Yes	Yes	Yes	Yes	Yes	Yes	Yes	Yes
9. Was the statistical significance assessed?	Yes	Yes	Yes	Yes	Yes	Yes	Yes	Yes	Yes	Yes	Yes	Yes	Yes	Yes	Yes	Yes	Yes
10. Are confidence intervals given for the main results?	No	No	No	Yes	No	No	Yes	No	Yes	No	No	Yes	No	Yes	No	No	Yes
11. Could there be confounding factors that have not been accounted for?	Cannot tell	Cannot tell	No	Cannot tell	Cannot tell	Cannot tell	Cannot tell	Cannot tell	Cannot tell	Cannot tell	Cannot tell	Cannot tell	Cannot tell	Cannot tell	Cannot tell	Cannot tell	Cannot tell
12. Can the results be applied to our organization?	Yes	Yes	Yes	Yes	Yes	Yes	Yes	Yes	Yes	Yes	Yes	Yes	Yes	Yes	Yes	Yes	Yes
Criteria met in the appraisal of the quality	9	9	11	10	9	10	10	8	10	7	9	9	10	9	9	10	8

### 2.5 Data extraction and analysis

Specifying information of the publication, the purpose of the study, study subjects, study context, methodology and statistically significant outcomes reported in each included article were extracted to the matrix and are listed in Table 3. The articles were analysed by narrative synthesis (Popay et al., 2006; Ryan, 2013). First, each article was read through once to form an overview of its content. They were then read repeatedly to obtain deeper insight into their content. Descriptions of all identified leadership styles were reduced into codes by both researchers independently. These codes were compared, checked and completed together to reach all the essential expressions from the data. The codes were compared according to their similarities and differences. Similar expressions were grouped under the same theme and the themes were named according to associated content. For example, in the study by Majeed and Fatima (2020) the exploitative leadership style was described as ‘egoistic behaviour, manipulation and pressurizing’. This code was grouped under the theme ‘nurse leaders’ selfishness and nurses' bad treatment’ and categorized under the core theme ‘destructive’. Altogether three core themes were identified (destructive, supportive and relationally focused leadership styles). Then the measurements of work‐related well‐being and reported relationships (direct and indirect) between leadership styles and work‐related well‐being were examined. Due to the heterogeneity of the leadership styles and measurements of work‐related well‐being, further synthesis such as meta‐analyses of the data was not possible (Higgins & Green, 2011).

**TABLE 3:** Overview of the selected articles

Research, Author (year), country, aim/design	Participants, study context	Methods	Statistically significant results
Bobbio et al. (2012), Italy To test the impact of perceived empowering leadership style expressed by the nurse supervisor, nurses' perceived organizational support, trust in the leader, and trust in the organization on nurses' job burnout *Design* Cross‐sectional study	From a total of 482 questionnaires, 273 were received from the nursing staff of a public general hospital in Italy Response rate: 56.62%	*Measurements* The Empowering Leadership Questionnaire, Italian version: 38 items The Survey of Perceived Organizational Support: 8 items The Organizational Trust Inventory: reduced 12‐item version The Maslach Burnout Inventory‐General Survey (MBI‐GS): 16 items *Data analyses* LISREL 8.80. Cronbach's alpha, descriptive statistics, Pearson's correlation coefficients, *t* test, mean scores	Empowering leadership showed a high positive effect on trust in the leader. Trust in the leader showed a negative impact on nursing staff's emotional exhaustion and cynicism dimensions of job burnout. All the empowering leadership factors were negatively correlated with job burnout, emotional exhaustion (average *r* = −.30) and cynicism (average *r* = −.27). Trust in the leader was negatively correlated with job burnout, emotional exhaustion (*r* = −.38, *P* < .01) and cynicism (*r* = −.30, *P* < .01), but did not significantly correlate with reduced accomplishment (*r* = .04)
Bobbio and Manganelli (2015), Italy To link perceived leadership style, perceived organizational support, trust in the leader and in the organization, job burnout among nurses and their subsequent intention to leave the hospital *Design* Cross‐sectional survey	From a total of 1743 questionnaires, 711 were received from nurses from two large Italian public hospitals (sample 1 Veneto Region and sample 2 Puglia Region) Response rates: 41% hospital 1 and 40.5% hospital 2	*Measurements* Servant Leadership Survey: 30 items on their nurse managers' behaviours on the eight dimensions of the servant leadership survey, such as empowerment, accountability, standing back, humility, authenticity, courage, forgiveness and stewardship Survey of perceived organizational support: 3 items from Italian version of Organizational Trust Inventory Italian version of MBI‐general Survey: 16 items on job burnout Intention to leave the organization: 3 items *Data analyses* Correlational analyses and structural equation modelling (SEM) analyses	Levels of job burnout, emotional exhaustion and cynicism could be considered moderate or low, whereas professional efficacy scores were high For the job burnout factors, the servant leadership score was, in sample 1, linearly independent from emotional exhaustion (−.07), negatively correlated to cynicism (−.18, *P* < .01) and positively associated with professional efficacy scores (.16, *P* < .01). In sample 2, servant leadership was negatively correlated with emotional exhaustion (−.30, *P* < .01) and cynicism (−.23, *P* < .01) and positively associated with professional efficacy (.11, *P* < .05) Trust in the leader was negatively correlated with emotional exhaustion (−.22, *P* < .01 in sample 1, −.30, *P* < .01 in sample 2) and cynicism (−.25, *P* < .01 in sample 1, −.22, *P* < .01 in sample 2). Trust in the leader showed a positive relation with professional efficacy (.16, *P* < .01 in sample 1, .14, *P* < .05 in sample 2) The standardized indirect effects of servant leadership on the three burnout factors vie the mediation of trust in the leader, and of servant leadership on intention to leave via the mediation of both trust in the leader and the cynicism factor of burnout, were statistically significant and equal to −.09 (*P* < .01), .14 (*P* < .01), −.16 (*P* < .01) and −.03 (*P* < .01), respectively, in sample 1 and equal to −.14 (*P* < .01), .13 (*P* < .01). −.16 (*P* < .01) and .04 (*P* < .01) in sample 2
Ebrahimzade et al. (2015), Iran To investigate the relationship between nursing managers' leadership styles and nurse burnout *Design* Cross‐sectional survey, descriptive correlational study	230 nurses working at hospital, 207 returned the questionnaires Response rate: 90%. Male (*n* = 37) and female (*n* = 170)	*Measurements* A questionnaire of demographic characteristics The Maslach Burnout Inventory: 22 items on emotional exhaustion, depersonalization and reduced personal accomplishment The Multifactor Leadership Questionnaire (MLQ): 36 questions on transformational, transactional, and laissez‐faire leadership styles *Data analyses* SPSS 19 used for independent *t*‐tests, one‐way analysis of variance, and Pearson's correlation analysis	Participants scored on the dimensions of burnout: 27.26 (moderately high), 5.96 (low), and 30.38 (high) for emotional exhaustion, depersonalization, and reduced personal accomplishment, respectively Transformational (*r* = −.150, *P* = .03) and transactional leadership (*r* = −.135, *P* = .04) both had significant negative relationships with total burnout. Emotional exhaustion was related negatively to transformational (*r* = −.172, *P* = .013) and transactional leadership (*r* = −.165, *P* = .018) and depersonalization to transformational (*r* = −.158, *P* = .023) and transactional leadership (*r* = −.204, *P* = .003). Laissez‐faire leadership style had a significant negative relationship with reduced personal accomplishment (*r* = −.199, *P* = .004)
Kaffashpoor and Sadeghian (2020), Iran To investigate the connection between ethical leadership and the perceived wellbeing with job satisfaction as mediator, among nurses *Design* Descriptive‐correlational survey	166 nurses from a total of 730 nurses from private hospitals in Mashhad. Response rate: 83%	*Measurements* Questionnaire included: 4 job satisfaction items 9 ethical leadership items 4 subjective wellbeing items *Data analyses* SPSS24 Smart PLS3 Pearson correlation coefficients, Structural Equation Modelling (SEM)	Ethical leadership had a significant effect (*β* = .155, *T*‐value = 2.420) on subjective wellbeing, and indirect effect via job satisfaction (*β* = .286, *T*‐value = 7.160) on subjective wellbeing (*β* = .619, *T*‐value = 11.338)
Laschinger et al. (2013), Canada To examine effects of authentic leadership and structural empowerment on the emotional exhaustion and cynicism of new graduate nurses and experienced acute‐care nurses *Design* Cross‐sectional study	342 from a total of 907 new graduate nurses (less than 2 years of practice experience) and 273 from 600 experienced nurses Response rates: 37.7% for new graduate nurses (91.5% female) and 48% for experienced nurses (93.4% female)	*Measurements* The Conditions of Work Effectiveness Questionnaire‐II (CWEQ‐II): 12 items on the four components of structural empowerment (opportunity, information, support and resources) The Authentic Leadership Questionnaire (ALQ): 16 items on the four components of nurses' perception of managers' authentic leadership (relational transparency, moral/ethical conduct, balanced processing and self‐awareness) The Maslach Burnout Inventory‐General Survey: 10 items to measure two components of burnout (emotional exhaustion and cynicism) *Data analyses* SPSS 19.0. for Windows (SPSS Inc., Chicago, IL, USA). Descriptive statistics and SEM with AMOS version 19.0 software (SPSS Inc., Chicago, IL, USA)	Both groups reported that immediate managers had moderate levels of authentic leadership, with no significant differences between them, except that the new graduate nurses rated their leaders as more self‐aware (*t*_(613)_ = 2.23, *P* < .05) and reported significantly higher scores for access to opportunity (*t*_(613)_ = 2.33, *P* < .05), information (*t*_(613)_ = 5.20, *P* < .05) and resources (*t*_(613)_ = 3.33, *P* < .05). The experienced nurses reported significantly higher rates of emotional exhaustion (*t*_(613)_ = 2.38, *P* < .05), severely high levels on average (mean = 3.20) according to Maslach et al.'s criteria Authentic leadership was positively related to empowerment in both groups (experienced nurses *β* = .411, *P* < .001 and new graduates *β* = .402, *P* < .001). Authentic leadership had a small negative effect on cynicism (experienced nurses *β* = −.101, *P* < .001, new graduates *β* = −.125, *P* < .001). Higher levels of authentic leadership and structural empowerment were associated with lower emotional exhaustion and cynicism in both groups. Indirect effects of authentic leadership on emotional exhaustion (*β* = −.129, *P* = .001 experienced nurses, *β* = −.066, *P* = .002 new graduates) and cynicism (*β* = −.147, *P* = .001 experienced nurses, *β* = −.118, *P* = .001 new graduates) and structural empowerment on cynicism (*β* = −.210, *P* ≤ .001 experienced nurses, *β* = −.084, *P* = .003 new graduates) were significant in both groups, but were larger in the experienced nurse group
Laschinger et al. (2014), Canada To examine effects of resonant leadership and empowerment on nurses' experiences of workplace incivility and burnout and job satisfaction *Design* Cross‐sectional study	From a total of 3600 nurses, 1241 returned the questionnaire Response rate: 35% (93.6% female)	*Measurements* The Resonant Leadership Scale: 10 items on resonant leadership behaviours of the current supervisor Global Empowerment Scale: two items on workplace empowerment The Workplace Incivility Scale: Employees' self‐reported exposure to co‐worker incivility in the past month The Maslach Burnout Inventory‐General Survey: five items to measure the core component of burnout, emotional exhaustion A 4‐item global measure of work satisfaction *Data analyses* SPSS 20.0, IBM, 2011. Descriptive, inferential and reliability analyses. SEM using Analysis of Moment Structures (AMOS) version 20.0 (IBM, 2011)	On average nurses did not rate their immediate supervisors highly for use of resonant leadership behaviours (M = 3.22) Nurses' levels of emotional exhaustion (M = 2.87) were just below Leiter & Maslach's cut‐off for severe burnout (>3.0). Resonant leadership had a strong positive direct effect on workplace empowerment (*β* = .47) and a significant direct effect on job satisfaction (*β* = .16) Resonant leadership also had both a direct influence on job satisfaction (*β* = .16) and an indirect effect through creating a greater sense of empowerment (*β* = .47) and subsequently lower incivility (*β* = −.25) and emotional exhaustion (*β* = −.38)
Laschinger and Fida (2014), Canada To investigate effects of authentic leadership and psychological capital on new graduate burnout, occupational satisfaction and workplace mental health over the first year of employment *Design* Follow up study	At T1 (2010) from a total of 907 newly graduated nurses, 342 participants returned the questionnaire. Response rate: 37.7% At T2 (2011) of the 342 participants who returned the questionnaire at T1, 205 returned questionnaires. Response rate: 59.9%	*Measurements* The Authentic Leadership Questionnaire: 16 items to measure four dimensions of authentic leadership behaviour (self‐awareness, moral‐ethical perspective, balanced processing and transparency) The Psychological Capital Questionnaire: 24 items to measure four dimensions (self‐efficacy, hope, optimism and resiliency) The Emotional Exhaustion and Cynicism subscales of the Maslach Burnout Inventory‐General Survey (MBI‐GS): both subscales contain 5 items Work Satisfaction: 4 items adapted from Shaver and Lacey Mental Health Index (MHI‐5): 5 items to measure depressive symptomatology *Data analyses* SPSS 16.0 and MPlus (version 7.1, Muthen & Muthen 1998–2010): Descriptive statistics, specific means, standard deviations, kurtosis and skewness, correlations, Cronbach's alpha, Chi‐square, comparative fit index, the root mean square error of approximation and standardized root mean square residuals	Authentic leadership indirectly influenced mental health (*β* = −.10, 95% CI −.177 to −.026) and negatively influenced both burnout dimensions' intercepts (EE T1 M 2.75, SD 1.56, *β* = −.17, *P* < .05, CYN T1 M 1.64, SD 1.33, *β* = −.25, *P* < .05, EE T2 M 2.90, SD 1.53, *β* = −.22, *P* < .05, CYN T2 M 1.91, SD 1.42, *β* = −.22, *P* < .05)
Laschinger et al. (2015), Canada To link authentic leadership, areas of worklife, occupational coping self‐efficacy, burnout and mental health among new graduate nurses *Design* Cross‐sectional survey	From a total of 3743 new graduate nurses, 1009 (92.5% female) returned the questionnaire Response rate: 27%	*Measurements* The Authentic Leadership Questionnaire: 16 items to measure four dimensions of authentic leadership behaviour (self‐awareness, moral‐ethical perspective, balanced processing and transparency) Areas of Worklife Scale: 18 items to measure six dimensions of the work environment that contribute to employees' experience (workload, control, reward, a sense of community, fairness and values congruence) Occupational Coping Self‐Efficacy Scale: 9 items to measure the extent to which respondents believe that they would cope with stressful occupational situations The Maslach Burnout Inventory‐General Survey: 5 items to measure emotional exhaustion and cynicism The depressive symptoms scale of the General Health Questionnaire: 12 items to measure mental health *Data analyses* SPSS 22.0 (IBM, 2014)	On average, the new graduate nurses rated their supervisors' authentic leadership behaviours as 2.60 out of 4.0 (SD 0.87). The nurses reported higher emotional exhaustion levels (3.24, SD 1.48), and cynicism (M 1.60, SD 1.56). Emotional exhaustion levels were in the severe burnout category according to norms presented by Schaufeli et al. (>3.0). Average rating of mental health was positive, 2.78 (SD 0.47). Authentic leadership was related to cynicism (−.25), emotional exhaustion (−.19) and mental health (.22). All hypothesized paths were significant at the *P* < .01 level. authentic leadership was found to have direct effects on areas of worklife (*β* = .50), which in turn had a direct effect on new graduates' burnout (*β* = −.41). Burnout subsequently had a negative effect on mental health (*β* = −.69). Indirect effects of authentic leadership on burnout and mental health were significant (*β* = .072 and *β* = .05)
Majeed and Fatima (2020), Pakistan To test the impact of exploitative leadership on nurses' psychological distress. To examine the mediating role of negative affectivity between exploitative leadership and psychological distress. To investigate the moderating role of psychological detachment from work on the relationship between exploitative leadership and negative affectivity *Design* Quantitative and causal study, questionnaires at three times with 15‐day intervals	Nurses from gynaecology, orthopaedics, otolaryngology, cardiology, nephrology and gastroenterology departments. Of 15 hospitals in Pakistan: four in Islamabad and Rawalpindi, five in Sargodha and six in Lahore At 1st, 2nd and 3rd times 346, 289 and 231 nurses responded, and their responses were used for data analysis. Response rate: 59.2%	*Measurements* 15‐item scale by Schmid et al. for exploitative leadership 10‐item scale by Watson et al. to measure negative affectivity 10‐item scale by Kessler et al. to measure psychological distress 4‐item scale by Sonnentag and Fritz to measure the psychological detachment from work *Data analyses* SPSS statistical package version 20. One‐way ANOVA, confirmatory factor analysis, mean, standard deviation, Pearson bivariate correlation, Cronbach alpha	Exploitative leadership was significantly and positively connected to negative affectivity (*r* = .48, *P* < .001) and psychological distress (*r* = .35, *P* < .001). Exploitative leadership causes psychological distress (*β* = .18, *P* < .001) and leads to a significant increase in negative affectivity among nurses (*β* = .50, *P* < .001). Negative affectivity mediates the relationship between exploitative leadership and psychological distress (*β* = .16). Exploitative leadership positively leads to psychological distress (*β* = .18, *P* < .001) and negative affectivity enhances psychological distress (*β* = .42, *P* < .001). Negative affectivity mediates the relationship between exploitative leadership and psychological distress (*β* = .22)
McKenna and Jeske (2021), Ireland To explore relationships between ethical leadership style and emotional exhaustion, work engagement, and turnover intention *Design* Cross‐sectional survey	89 Irish nurses from three Irish hospitals Response rate: 54.7%	*Measurements* 9‐item version of the Utrecht Work Engagement Scale 5‐item Decision Authority subscale from the LQWLQ‐N scale 10‐item Ethical Leadership Scale 9‐item Emotional Exhaustion subscale from the Maslach Burnout Inventory *Data analyses* SPSS 23. Descriptive, correlation. LISREL 9.20, path model The PROCESS procedure	Ethical leadership has a significant direct effect on emotional exhaustion (*β* = −.13, *t* = −2.27, *P* < .05) and indirect effect via decision authority (*β* = .41, *t* = 3.71, *P* < .05) on emotional exhaustion (*β* = −.10, *t* = .70, *P* = ns)
Munir et al. (2012), Denmark To explore the mediating effects of work‐life conflict on relationships between transformational leadership and job satisfaction and psychological wellbeing *Design* A longitudinal survey with baseline and 18‐month follow‐up	For baseline measures, 447 out of 551 questionnaires were completed. For follow‐up measures, 274 out of 521 were completed. In total, 188 participants from elderly care staff provided data at both baseline and follow‐up. Response rate: 53% Most staff were healthcare assistants (61%), 12% were nurses' 21% had other health‐related occupations and 8% had no healthcare‐related education	*Measurements* The Global Transformational Leadership Scale: 7 items on transformational leadership The Copenhagen psychosocial questionnaire (COPSOQ): 2 items on work‐life conflict A 5‐item job satisfaction scale A 5‐item psychological wellbeing scale Demographic measures: Age, gender, cohabitation, number of children living at home and tenure *Data analyses* SPSS 19.0 (SPSS Inc., Chicago, IL, USA) used for correlation and regression analyses	Transformational leadership was directly associated with work‐life conflict (*β* = −.29, *F*(6, 119) = 4.71, *P* < .05) and work‐life conflict mediated relationships between transformational leadership style and psychological well‐being (*β* = .20, *F*(6, 117) = 1.74, *P* < .05)
Nelson et al. (2014), Canada To investigate the relationship between authentic leadership, psychological well‐being at work and climate *Design* Cross‐sectional survey	Of the 7997 nurses contacted, 859 participated at T1 (response rate: 10.7%), and of these, 608 participated at T2 (response rate: 71.8%). The final sample consisted of 406 pairings	*Measurements* 12‐item Authentic Leadership Questionnaire 17‐item Perceptions of Work Climate scale adapted from scale developed by Roy (1989) 25‐item Psychological well‐being at work scale adapted from scale developed by Masse et al. (1998) Demographic variables *Data analyses* PROCESS, SPSS 21.0 Descriptive statistics, assessment of normality, correlations	There was a connection between authentic leadership style and psychological well‐being at work. Authentic leadership style had indirect association via work climate (.58, *P* < .001, *R* ^2^ = .30) with psychological well‐being at work (.23, *P* < .001, *R* ^2^ = .22)
Pishgooie et al. (2018), Iran To explore relationships between leadership styles and nurse job stress and anticipated turnover *Design* Cross‐sectional study	The estimated required sample size was 1548.34 subjects and 1703 nurses were recruited through simple random sampling. In total, 1617 questionnaires were completed and returned (72.2% female)	*Measurements* Demographic questionnaire: 9 items Multifactor Leadership Questionnaire (MLQ) 5X: 45 items on leadership style Health and Safety Executive Questionnaire (HSE): 35 items on job stress Anticipated Turnover Scale (ATS): 12 items *Data analyses* SPSS 20 (SPSS Inc., Chicago, IL, USA) used for correlation analyses, Kolmogorov–Smirnov tests and calculations of frequencies, percentages, and measures of central tendency and dispersion	Dominant leadership style used by the head nurses was transactional (2.47 ± 0.86). Significant negative correlations between transformational and transactional leadership styles and job stress were found, plus significant positive correlations between laissez‐faire leadership style, job stress and anticipated turnover, as well as between job stress and anticipated turnover (all *P* < .001). A positive correlation between transformational leadership style and anticipated turnover, but negative correlation between transactional leadership style and anticipated turnover (*r* = −.28, *P* ≤ .001) were also found. Transformational, transactional and laissez‐faire leadership styles all had direct associations with job stress (*r* = −.34, *P* < .001; *r* = −.44, *P* < .001; and *r* = .23, *P* < .001, respectively
Read and Laschinger (2015), Canada To test a model linking authentic leadership of managers to new graduate nurses' perceptions of structural empowerment, relational social capital, mental health and job satisfaction *Design* A longitudinal survey	At time 1 (2010), 709 nurses in acute care hospitals who had graduated in the last 2 years, of whom 342 returned surveys (response rate: 48%), at time 2 (2011) 342 nurses, and a total of 191 matched useable returns were received (response rate: 55.8%). Male (*n* = 17) and female (*n* = 174)	*Measurements* The Authentic Leadership Questionnaire (ALQ): 16 items divided into relational transparency, balance processing, self‐awareness and internalized moral perspective The Conditions of Work Effectiveness Questionnaire II (CWEQ‐II): access to opportunity, information, support and resources (three items each) The Sense of Community subscale of the Areas of Worklife Scale (AWS): five items assessing individuals' perceptions of social relationships in the workplace The Mental Health Inventory (MHI‐5), a five‐item scale measuring experienced frequency of negative mental health symptoms during the last month 4‐item scale of job satisfaction *Data analyses* SPSS (version 21.0, IBM 2012) and Amos software (version 21.0, IBM 2012) used for chi‐square tests, paired *t*‐tests, and SEM maximum likelihood estimation	At time 1, nurses reported that their manager often demonstrated authentic leadership behaviours with a mean score of 2.47 (.88) out of 4. At time 2, few nurses reported experiencing frequent mental health symptoms: Average rating 2.39 (.99) out of 6 Authentic leadership had a significant positive effect on structural empowerment (*β* = .50, *P* < .05), which in turn had a significant effect on relational social capital (*β* = .70, *P* < .05). Relational social capital had a significant negative effect on mental health symptoms (*β* = −.21, *P* < .05) Analysis of indirect effects showed that authentic leadership had a significant effect on relational social capital through structural empowerment (*β* = .35, *P* < .05), and significant effects on mental health symptoms (*β* = −.07, *P* = .017) through its effects on structural empowerment and relational social capital
Rodwell and Munro (2013), Australia To investigate relationships between three types of organizational resources and the impact of job demands on nurse's well‐being and attitudes towards their work *Design* Cross‐sectional survey	From a total of 771 employees, 273 nurses and midwives completed the survey Response rate: 35.4%	*Measurements* A global 6‐item job satisfaction scale developed by Agho et al. 8‐item affective organizational commitment scale developed by Allen and Meyer The General Health Questionnaire‐12 (GHQ‐12) 10‐item NA scale from the Positive and Negative Affectivity Scale (PANAS) 11‐item scale of job demands developed by Caplan et al. 9‐item scale of job control developed by Karasek 4‐item social support scale developed by Caplan et al. 20‐item organizational justice scale developed by Colquitt *Data analyses* SPSS 18.0 for Windows (SPSS Inc. 2009) used for multiple regression analyses. Descriptive and inferential analyses	Supervisor support was significantly associated with well‐being (*β* = .18, *P* < .05)
Sabbah et al. (2020), Lebanon To assess the leadership styles of nurse leaders. To explore the connection between the perceived style and the quality of life of the nurses *Design* Cross‐sectional survey	250 nurses from eight hospitals in Tripoli and Saida Response rate: 96.1%	*Measurements* Characteristics 45‐item Multifactor Leadership Questionnaire 5X Short Form (MLQ 5X Short Form) 36‐item SF‐12v2 Health Survey (SF‐12v2). *Data analyses* SPSS 22.0 (IBM SPSS Statistics, USA). Frequencies, percentage, mean, median, standard deviation, ANOVA, Spearman's correlation SF scoring software V5 setup	Transformational leadership style and transactional leadership styles were positively and highly correlated with outcome factors (extra efforts, satisfaction and effectiveness) (*P* < .001), whereas passive/avoidant leadership style correlated negatively and weakly with extra efforts and moderately with effectiveness and satisfaction (*P* < .001). Transformational and transactional leadership styles had direct association with mental health (*r* = .20, *P* < .001/*r* = .24, *P* < .001) and physical health (*r* = .15, *P* < .001/*r* = .17, *P* < .001). Passive/avoidant leadership styles had direct association with mental health (*r* = −.13, *P* < .05)
Trepanier et al. (2019), Canada To investigate the relationship between tyrannical and laissez‐faire leadership styles and nurses' work‐related well‐being *Design* Cross‐sectional survey	2500 nurses were contacted and 399 participated Response rate: 16%	*Measurements* 8‐item Destructive Leadership Scale 9‐item Psychological Need Thwarting Scale 8‐item Multidimensional Work Motivation Scale 10‐item Maslach Burnout Inventory General Survey 6‐item Occupational Commitment Questionnaire 4‐item Job Performance Scale adapted from the In‐Role Performance Subscale of the Organizational Citizenship Behaviour Scale *Data analyses* Mplus, structural equation modelling	Tyrannical leadership was positively related to frustration of autonomy, relatedness and competence. Laissez‐faire leadership was positively related to frustration of autonomy. Low‐quality motivation positively predicted burnout and negatively predicted affective commitment and job performance. Laissez‐faire leadership was also a direct negative predictor of affective commitment and positive predictor of burnout Laissez‐faire leadership had direct association with burnout (.20, *P* < .01) and indirect association via need frustration – Autonomy NFA (.16, *P* < .05) to controlled motivation through NFA (.27, *P* < .01) with burnout (.68, *P* < .01) Tyrannical leadership style had indirect association via need frustration – Autonomy NFA (.26, *P* < .01) and need frustration – Competence NFC (.22, *P* < .01) to controlled motivation through NFA (.27, *P* < .01) and NFC (.60, *P* < .01) with burnout (.68, *P* < .01)

## 3 RESULTS

### 3.1 Characteristics of the studies

The studies (*N* = 17) were conducted in eight countries: Italy (*n* = 2), Canada (*n* = 7), Iran (*n* = 3), Australia (*n* = 1), Pakistan (*n* = 1), Ireland (*n* = 1), Lebanon (*n* = 1) and Denmark (*n* = 1). The studies were cross‐sectional and longitudinal follow‐up surveys. Collectively, they described 12 leadership styles. In total, 11 instruments were used in them to measure work‐related well‐being. The number of participants per study ranged from 89 to 1617 and included: non‐graduated, newly graduated and experienced nurses; healthcare assistants; midwifes; and other health professionals such as physiotherapists or non‐healthcare personnel (e.g. cleaning staff). Participants worked in elderly care facilities and acute or critical care, in public, private and non‐profit maternity or governmental hospitals (Table 3).

### 3.2 Destructive leadership styles and nurses' work‐related well‐being

Five studies described destructive leadership styles, such as management‐by‐exception (Sabbah et al., 2020), passive/avoidant including laissez‐faire (Ebrahimzade et al., 2015; Pishgooie et al., 2018; Sabbah et al., 2020; Trepanier et al., 2019), exploitative (Majeed & Fatima, 2020) and tyrannical (Trepanier et al., 2019) styles (Table 4). Common described theme of these styles were nurse leaders' selfishness and nurses' bad treatment. Leaders with laissez‐faire leadership styles relied without justification on their employees' decision‐making skills and provided them too much autonomy. They avoided decision‐making and ignored leader's responsibilities (Ebrahimzade et al., 2015; Pishgooie et al., 2018). Characteristic features of exploitative leadership included egoistic behaviour, manipulation, belittling and pressuring employees (Majeed & Fatima, 2020). Tyrannical leaders lessened, downplayed and diminished their subordinates, often had unreasonable expectations and tended to meet goals of the organization at the cost of their employees' well‐being (Trepanier et al., 2019).

**TABLE 4:** The statistically significant associations between leadership styles and work‐related well‐being

Leadership style/reference	Measure of work‐related well‐being	Direct associations with work‐related well‐being	Mediating variable for indirect associations	Indirect associations to work‐related well‐being
**Destructive leadership styles**				
***Management‐by‐exception***				
Sabbah et al. (2020)	Health Survey (SF‐12v2)	**Mental health** (*r* = −.13, *P* < .05)		
***Laissez‐faire***				
Sabbah et al. (2020)	Health Survey (SF‐12v2)	**Mental health** (*r* = −.13, *P* < .05)		
Ebrahimzade et al. (2015)	Maslach Burnout Inventory General Survey (MBI)	**Reduced personal accomplishment** (*r* = −.199, *P* = .004)		
Pishgooie et al. (2018)	Health and Safety Executive Questionnaire (HSE)	**Job stress** (*r* = .23, *P* < .001)		
Trepanier et al. (2019)	Maslach Burnout Inventory General Survey (MBI)	**Burnout** (.20, *P* < .01)	Via **need frustration – Autonomy NFA** (.16, *P* < .05) To **controlled motivation through NFA** (.27, *P* < .01)	**Burnout** (.68, *P* < .01)
***Exploitative***				
Majeed and Fatima (2020)	Scale developed by Kessler et al. Scale developed by Sonnentag and Fritz	**Psychological distress** (*β* = .18, *P* = .004)	**Negative affectivity** (*β* = .50, *P* < .001)	**Psychological distress** (*β* = .33, *P* < .001)
***Tyrannical***				
Trepanier et al. (2019)	Maslach Burnout Inventory General Survey (MBI)		Via **need frustration – Autonomy NFA** (.26, *P* < .01) **A**nd **need frustration – Competence NFC** (.22, *P* < .01) To **controlled motivation through NFA** (.27, *P* < .01) and NFC (.60, *P* < .01)	**Burnout** (.68, *P* < .01)
**Supportive leadership styles**				
***Supportive***				
Rodwell and Munro (2013)	General Health Questionnaire (GHQ)	**Psychological health** (*β* = .18, *P* < .05)		
***Transactional***				
Ebrahimzade et al. (2015)	Maslach Burnout Inventory General Survey (MBI)	**Total burnout** (*r* = −.135, *P* = .04) **Emotional exhaustion** (*r* = −.165, *P* = .018) **Depersonalization** (*r* = −.204, *P* = .003)		
Pishgooie et al. (2018)	Health and Safety Executive Questionnaire (HSE)	**Job stress** (*r* = −.44, *P* < .001)		
Sabbah et al. (2020)	Health Survey (SF‐12v2)	**Physical health** (*r* = .17, *P* < .001) **Mental health** (*r* = .24, *P* < .001)		
***Empowering***				
Bobbio et al. (2012)	Maslach Burnout Inventory General Survey (MBI)		**Trust in leader** (*R* ^2^ = .67) **Trust in the organization** (*R* ^2^ = .37)	**Emotional exhaustion** (*r* = −.38, *P* < .01) **Cynicism** (*r* = −.30, *P* < .01) **Reduced professional accomplishment** (*r* = .04)
***Resonant***				
Laschinger et al. (2014)	Maslach Burnout Inventory General Survey (MBI)		**Empowerment** (*β* = .47, *P* < .05)	**Emotional exhaustion** (*β* = −.20, *P* < .05)
**Relationally focused leadership styles**				
***Transformational***				
Ebrahimzade et al. (2015)	Maslach Burnout Inventory General Survey (MBI)	**Total burnout** (*r* = −.150, *P* = .031) **Emotional exhaustion** (*r* = −.172, *P* = .013) **Depersonalization** (*r* = −.158, *P* = .023)		
Pishgooie et al. (2018)	Health and Safety Executive Questionnaire (HSE)	**Job stress** (*r* = −.34, *P* < .001)		
Sabbah et al. (2020)	Health Survey (SF‐12v2)	**Physical health** (*r* = .15, *P* < .001) **Mental health** (*r* = .20, *P* < .001)		
Munir et al. (2012)	5‐item psychological well‐being scale		**Work‐life conflict** (*β* = −.29, *F*(6, 119) = 4.71, *P* < .05)	**Psychological well‐being** (*β* = .20, *F*(6, 117) = 1.74, *P* < .05)
***Authentic***				
Laschinger et al. (2013)	Maslach Burnout Inventory General Survey (MBI‐GS)	**Cynicism** *β* = −.101, *P* < .001 for experienced nurses and *β* = −.125, *P* < .001 for new graduates	**Structural empowerment**, experienced nurses (*β* = .411, *P* < .001) and new graduate nurses (*β* = .402, *P* < .001)	**Emotional exhaustion** (*β* = −.129, *P* = .001) for experienced nurses and for new graduate nurses (*β* = −.066, *P* = .002) **Cynicism** (*β* = −.147, *P* = .001) for experienced nurses and for new graduate nurses (*β* = −.118, *P* = .001)
Nelson et al. (2014)	Psychological well‐being at work scale adapted from a scale by Masse et al.		**Work climate** (.58, *P* < .001, *R* ^2^ = .30)	**Psychological well‐being at work** (.23, *P* < .001, *R* ^2^ = .22)
Laschinger and Fida (2014)	Maslach Burnout Inventory General Survey (MBI‐GS) Psychological Capital Questionnaire Mental Health Index (MHI‐5)		**Emotional exhaustion** (*β* = −.23)	**Mental health problems** (*β* = −.10)
Laschinger et al. (2015)	Maslach Burnout Inventory General Survey (MBI‐GS) General Health Questionnaire (GHQ)		**Areas of worklife** (*β* = .50, *P* < .01) with direct effect on occupational coping self‐efficacy (*β* = −.35, *P* < .01) and burnout (*β* = −.41, *P* < .01)	**Burnout** (*β* = −.072, *P* < .01) **Mental health** (*β* = .050, *P* < .01)
Read and Laschinger (2015)	Mental Health Index (MHI‐5)		**Relational social capital through structural empowerment** (*β* = .35 *P* < .05)	**Mental health symptoms** (*β* = −.07 *P* < .05)
***Servant***				
Bobbio and Manganelli (2015)	Maslach Burnout Inventory General Survey (MBI)		**Trust in leader** *R* ^2^ = .63, *P* < .01 (sample 1, Veneto region) *R* ^2^ = .64, *P* < .01 (sample 2, Puglia region)	**Emotional exhaustion**, *R* ^2^ = .07, *P* < .01 (sample 1, Veneto region), *R* ^2^ = .07, *P* < .01 (sample 2, Puglia region) **cynicism**, *R* ^2^ = .06, *P* < .01 (sample 1, Veneto region), *R* ^2^ = .05, *P* < .01 (sample 2, Puglia region) **Professional efficacy**, *R* ^2^ = .02, *P* < .01 (sample 1, Veneto region), *R* ^2^ = .02, *P* < .01 (sample 2, Puglia region)
***Ethical***				
McKenna and Jeske (2021)	Maslach Burnout Inventory General Survey (MBI)	**Emotional exhaustion** (*β* = −.13, *t* = −2.27, *P* < .05)	**Decision authority** (*β* = .41, *t* = 3.71, *P* < .05)	**Emotional exhaustion** (*β* = −.10, *t* = .70, *P* = ns)
Kaffashpoor and Sadeghian (2020)	A four subjective well‐being item scale	**Subjective wellbeing** (*β* = .155, *T*‐value = 2.420)	**Job satisfaction** (*β* = .462, *T*‐value = 7.445)	**Subjective wellbeing** (*β* = .619, *T*‐value = 11.338)

Associations of these destructive leadership styles with work‐related well‐being were measured by five scales: the Maslach Burnout Inventory General Survey (MBI‐GS) in two studies (Ebrahimzade et al., 2015; Trepanier et al., 2019), the Health and Safety Executive Questionnaire (HSE) by Pishgooie et al. (2018), the SF‐12v2 Health Survey by Sabbah et al. (2020) and scales developed by Kessler et al. and Sonnentag and Fritz in the study by Majeed and Fatima (2020).

Laissez‐faire leadership was found to have a direct significant negative relationship with personal accomplishment by Ebrahimzade et al. (2015), a positive significant correlation with job stress by Pishgooie et al. (2018), and a direct significant association with burnout by Trepanier et al. (2019). Passive/avoidant leadership styles negatively correlated with mental health according to Sabbah et al. (2020). Exploitative leadership style had both direct and indirect (through increases in negative affectivity) effects on employees' psychological distress, according to Majeed and Fatima (2020). Trepanier et al. (2019) also found that tyrannical leadership indirectly affected nurses' burnout through controlled motivation (Trepanier et al., 2019) (Table 4).

### 3.3 Supportive leadership styles and nurses' work‐related well‐being

Six studies described supportive leadership styles, such as supportive (Rodwell & Munro, 2013), transactional (Ebrahimzade et al., 2015; Pishgooie et al., 2018; Sabbah et al., 2020), empowering (Bobbio et al., 2012) and resonant (Laschinger et al., 2014). Common recognized themes of these leadership styles were faith in employees' resources, organizational procedures targeting to enhance employees' capacities, desire to utilize resources fully in prevailing circumstances and acting as an example. Transactional leaders were described as task‐oriented and to emphasize the roles of employees. They were said to concentrate on their relationships with their employees and promote interactions by building commitment to the organization. They encouraged employees by implementing performance‐based reward systems with penalties for deviating from standard action (Ebrahimzade et al., 2015; Pishgooie et al., 2018; Sabbah et al., 2020). Besides this, empowering and resonant leaders lead by example. They were described as participative in their decision‐making. They coached, encouraged and informed their employees and showed them concern. They took account of the current situation, human resources, emotions and the surroundings (Bobbio et al., 2012; Laschinger et al., 2014).

These supportive leadership styles' associations with work‐related well‐being were measured by four instruments: the MBI‐GS in three studies (Bobbio et al., 2012; Ebrahimzade et al., 2015; Laschinger et al., 2014), the HSE by Pishgooie et al. (2018), the General Health Questionnaire (GHQ) by Rodwell and Munro (2013) and the SF‐12v2 by Sabbah et al. (2020). Supervisor support significantly and directly predicted nurses' well‐being according to Rodwell and Munro (2013). Transactional leadership had a direct significant negative relationship with burnout, including emotional exhaustion and depersonalization (Ebrahimzade et al., 2015), and significant negative correlation with job stress (Pishgooie et al., 2018). Empowering leadership had strong positive effects on trust in both the leader and organization, and they mediated negative correlations of empowering leadership with elements of job burnout, specifically emotional exhaustion and cynicism (Bobbio et al., 2012). According to Laschinger et al. (2014), resonant leadership style had a strong positive direct influence on empowerment and thereby reduced incivility and emotional exhaustion (Table 4).

### 3.4 Relationally focused leadership styles and nurses' work‐related well‐being

In total, 12 studies described relationally focused leadership styles, such as transformational (Ebrahimzade et al., 2015; Munir et al., 2012; Pishgooie et al., 2018; Sabbah et al., 2020), authentic (Laschinger et al., 2013, 2015; Laschinger & Fida, 2014; Nelson et al., 2014; Read & Laschinger, 2015), servant (Bobbio & Manganelli, 2015) and ethical (Kaffashpoor & Sadeghian, 2020; McKenna & Jeske, 2021). These leadership styles differed from the leadership styles that we named supportive by the characteristics of the leader employee relationship and by the way leaders enhanced the growth of their employees and motivated them to involve in decision‐making. Common identified themes of the relationally focused leadership styles included desire to form an equal and reciprocal relationship with employees, challenging employees to participate and value‐driven behaviour. For example, transformational leaders were described as being visionary, intellectually stimulating and applying innovative methods to motivate their followers in problem‐solving. They successfully coached groups to achieve their goals, encouraged their employees to participate, and inspired their self‐confidence by giving them responsibilities and considering their personal differences (Ebrahimzade et al., 2015; Munir et al., 2012; Pishgooie et al., 2018; Sabbah et al., 2020). Ethical, authentic and servant leaders allowed employees to participate in decision‐making, clarified expectations, communicated openly and clearly, and encouraged them to flourish and learn from mistakes. They requested insights from employees before making important decisions and were described as honest, caring, fair, accountable, trustworthy and principled. They were aware of their own strengths and weaknesses, and personally integrated. (Bobbio & Manganelli, 2015; Kaffashpoor & Sadeghian, 2020; Laschinger et al., 2013; Laschinger & Fida, 2014; Laschinger et al., 2015; McKenna & Jeske, 2021; Read & Laschinger, 2015).

In the studies on relationally focused leadership styles work‐related well‐being was measured using eight instruments: the MBI‐GS in six studies (Bobbio & Manganelli, 2015; Ebrahimzade et al., 2015; Laschinger et al., 2013, 2015; Laschinger & Fida, 2014; McKenna & Jeske, 2021) and the Mental Health Index (MHI‐5) in two studies (Laschinger & Fida, 2014; Read & Laschinger, 2015). The HSE was used by Pishgooie et al. (2018), a 5‐item psychological well‐being scale by Munir et al. (2012), and a four subjective well‐being item scale by Kaffashpoor and Sadeghian (2020). In addition to these, the relationally focused leadership styles were measured by the Psychological Capital Questionnaire (Laschinger & Fida, 2014), the SF‐12v2 (Sabbah et al., 2020) and the Psychological Well‐being at Work scale, adapted from a scale by Masse et al. (Nelson et al., 2014). Transformational, ethical, servant and authentic leadership styles were found to have direct associations with work‐related well‐being. Transformational leadership reportedly had statistically significant direct negative relationships with burnout symptoms, including emotional exhaustion and depersonalization (Ebrahimzade et al., 2015) and a negative correlation with job stress (Pishgooie et al., 2018). Authentic leadership had a direct effect on psychological well‐being according to Nelson et al. (2014) and a small negative impact on cynicism (Laschinger et al., 2013), whereas ethical leadership had been found to have direct effects on subjective wellbeing (Kaffashpoor & Sadeghian, 2020) and emotional exhaustion (McKenna & Jeske, 2021) (Table 4).

In addition to direct associations with work‐related well‐being, transformational, authentic and ethical leadership styles also reportedly had indirect associations. Work‐life conflict mediated connections between transformational leadership and psychological well‐being (Munir et al., 2012). Empowerment (Laschinger et al., 2013) and work climate (Nelson et al., 2014) mediated connections between authentic leadership and psychological well‐being, including lower emotional exhaustion and cynicism, whereas job satisfaction was a mediator of effects of ethical leadership on subjective well‐being (Kaffashpoor & Sadeghian, 2020). Recorded mediators of authentic leadership effects included emotional exhaustion on mental health problems (Laschinger & Fida, 2014), work life on burnout and mental health (Laschinger et al., 2015) and relational social capital via structural empowerment on mental health symptoms (Read & Laschinger, 2015). Bobbio and Manganelli (2015) found that trust in the leader mediated significant standardized indirect effects of servant leadership in three burnout dimensions. Finally, McKenna and Jeske (2021) found that the mediator of ethical leadership effect on emotional exhaustion was decision authority (Table 4).

## 4 DISCUSSION

### 4.1 Consideration of results

This systematic review of quantitative studies showed that nurse leaders' leadership styles significantly affected nurses' work‐related well‐being. The results were consistent although the studies had been carried out in eight culturally diverse countries. The articles included in the review described 12 leadership styles, and 11 instruments designed to measure elements of work‐related well‐being. The diversity of instruments highlights the complexity of the concept of work‐related well‐being. The most frequently studied leadership styles were relationally focused styles: transformational, authentic, ethical and servant, all of which were found to be positively associated with nurses' work‐related well‐being. Our review also included 11 studies that Cummings et al. (2018) did not consider, covered an additional two databases that Cummings et al. (2018) did not screen, and had a 3‐year longer timeframe, covering publications up to December 2020 rather than August 2017. Moreover, our review had a broader scope than a recent review by Long (2020), who concentrated only on authentic leadership style, and another by Adams et al. (2019), who focused on intensive care unit settings and leaders' behaviour in them.

Transformational leadership and its impact on employees' well‐being has been studied extensively in several professional fields, yielding similar results to those discussed here (Gilbert et al., 2017; Jacobs et al., 2013; Kara et al., 2013; Kelloway et al., 2012; Nielsen et al., 2009; Sudha et al., 2016). For example, in hospitality, transformative leadership reportedly has a stronger positive effect on employees' work‐related well‐being than transactional leadership (Kara et al., 2013). Kelloway et al. (2012) found that non‐transformational leadership styles were negatively connected with trust, which mediated effects on employees' psychological well‐being. These results and those analysed in this review highlight the importance of evaluating leaders' leadership styles when considering nurses' work‐related well‐being. The available data suggest that nurse leaders should be capable of using supportive, empowering, resonant, transformational, transactional, authentic, ethical and servant leadership styles.

All the destructive leadership styles have negative connections to nurses' work‐related well‐being, and each of them were described in one article, except the laissez‐faire style, which was addressed in three studies. This raises questions about why these leadership styles have been studied so rarely. The systematic review by Cummings et al. (2018) also identified two destructive leadership styles: dissonant leadership and management‐by‐exception with negative associations to nurses' work‐related well‐being based on two studies. Thus, it is important to determine how widely unsatisfactory leadership styles are used, as well as how they affect nurses' work‐related well‐being and other nursing sensitive outcomes. More studies are needed to provide knowledge on characteristics of these destructive leadership styles, how they develop, their connections to adverse outcomes, and ways to prevent their negative effects. However, some relevant results have been presented, for example Lavoie‐Tremblay et al. (2016) showed that abusive leadership styles negatively affect patient outcomes.

Work‐related well‐being has been mainly evaluated in terms of burnout, in keeping with previous findings of reviews by Awa et al. (2010), Häggman‐Laitila and Romppanen (2017), Romppanen and Häggman‐Laitila (2017), Van Wyk and Pillay‐Van Wyk (2010) and Westermann et al. (2014). The instruments used in the included studies have been used widely before and their psychometric properties have been deemed good. It should, however, be noted that results of this review are based on comparatively narrow definitions of work‐related well‐being (Buffer et al., 2013); future studies in this area should address other dimensions of the concept and their associations with nurse leaders' leadership styles. A more comprehensive measurement approach would deepen our knowledge of the association between leadership styles and the promotion of work‐related well‐being. Another limitation of this review stems from methodological approaches of the studies. Their results were based on self‐assessment of work‐related well‐being and response rates were low. In the future, objective measures such as sick leave rates and productivity indicators should also be included in the study designs. Studies were cross‐sectional or follow‐up surveys and although many of them were based on sophisticated multivariate statistical analyses and results of the studies were consistent, the strength of the evidence remains low. Intervention and longitudinal studies are therefore needed to obtain stronger evidence regarding associations between nurse leaders' leadership styles and nurses' work‐related well‐being. The studies should also be replicated in different settings. We need also research‐based evidence regarding interventions' content and approaches that can fruitfully develop nurse leaders' leadership styles. Although we have some evidence of interventions such as coaching, summits, mentoring or workshops for nurse leaders, more studies are needed to identify what kind of leadership interventions are the most effective and the study designs need to be stronger. It is also pivotal to acknowledge the contextuality of the leadership practices and the influence of the whole team and culture on the work‐related well‐being, not just the individual relationships between nurse leaders and nurses (Cummings et al., 2021).

The results of our review of indirect effects of leadership styles on work‐related well‐being and the mediating factors underline the complexity of the focal phenomena. Many factors may interactively influence experiences of nurses' work‐related well‐being and the complexity requires further attention. Our review also showed that leadership styles can influence diverse other important variables, such as nurses' job satisfaction (Bobbio & Manganelli, 2015; Kaffashpoor & Sadeghian, 2020; Munir et al., 2012), incivility (Laschinger et al., 2014), empowerment (Laschinger et al., 2013, 2014; Read & Laschinger, 2015), turnover (McKenna & Jeske, 2021; Pishgooie et al., 2018), early career burnout (Laschinger & Fida, 2014; Trepanier et al., 2019), affective commitment (Trepanier et al., 2019), psychological detachment from work (Majeed & Fatima, 2020), work climate (Nelson et al., 2014), worklife and the development of trustful relationships (Read & Laschinger, 2015). Organizations should concentrate on creating structurally empowering work environments (Laschinger et al., 2013), trusting relationships and positive workplace environment (Read & Laschinger, 2015) when developing leadership styles of their leaders. When these factors are developed in the interventions together with leadership styles, we can assume that the interactivity of the factors produce positive comprehensive effects on the work‐related well‐being. We need future studies to develop these many‐sided interventions and to evaluate their effectiveness by objective measurements (Cummings et al., 2021).

Appropriate leadership styles are essential for creating healthy work environments, promoting nurses' well‐being, and avoiding high turnover. In addition to these nurse workforce outcomes, nurse leaders' performance influences patient outcomes (Goedhart et al., 2017; Lavoie‐Tremblay et al., 2016; Wong et al., 2013). Healthcare organizations are complex systems and leaders must be comfortable using several different leadership styles in different situations. Leaders must also be aware of their own leadership styles and their effectiveness and the mediating factors in the work environment regarding employees' work‐related well‐being, and constantly seek to improve their own skills and work environment. This is greatly facilitated by the support of superiors. Leaders need more knowledge on how to identify the differences between the different leadership styles and how to apply supportive and relationally focused styles instead of destructive styles. They also need training on how to use different leadership styles in different situations. The training should be systematic, evidence‐based and cover all levels of the organization. It should also provide a deep understanding of the complexity of the phenomena.

### 4.2 Limitations

Several limitations of this study have already been highlighted, but some others should also be mentioned. The search process was conducted with an information specialist using database directories in efforts to ensure that the search was sufficiently systematic and extensive. The search terms and selection process have been described in detail above to allow repeatability. Several electronic databases were searched, and the results were complemented by reviewing reference lists of articles included in the final sample to minimize the likelihood of selection bias. The language selection criterion may have caused the exclusion of relevant studies published in languages other than English or Finnish. The grey literature was not taken into account in the review. This may have also increased the probability of bias when acquiring material to review. The reliability of the analysis was increased by using a matrix, in which the analysed articles are described in detail.

## 5 CONCLUSION

This systematic review of quantitative studies clearly showed that nurse leaders' leadership styles significantly influence nurses' work‐related well‐being. In total, 14 of the reviewed studies focused on 12 leadership styles with positive impacts on nurses. More research is required on unrecommended leadership styles, their prevalence and their impact on nurses' work‐related well‐being. Work‐related well‐being was mainly measured and defined in terms of burnout; this narrow definition prevents a holistic analysis of the relationship between leadership styles and work‐related well‐being. The strength of the evidence in this field appears to be low; intervention studies are needed to get stronger evidence regarding nurse leaders' leadership styles and their direct and indirect impacts on nurses' work‐related well‐being to inform healthcare policy and organizations, educators, and researchers. In the developing of intervention studies on work‐related well‐being, the results of the indirect effects and the mediating factors of the leadership styles should be acknowledged. Because nurse leaders' leadership styles affect nurses' work‐related well‐being, systematic evaluation of these styles in organizations is important. Organizations should invest in nurse leaders' education. Results of this review could be used when developing work environments, planning and implementing leadership training.

## CONFLICT OF INTERESTS

The authors declare no conflict of interests.

## Data Availability

The data that supports the findings of this study are available in the supplementary material of this article
